# RNA-guided transcriptional silencing in vivo with *S. aureus* CRISPR-Cas9 repressors

**DOI:** 10.1038/s41467-018-04048-4

**Published:** 2018-04-26

**Authors:** Pratiksha I. Thakore, Jennifer B. Kwon, Christopher E. Nelson, Douglas C. Rouse, Matthew P. Gemberling, Matthew L. Oliver, Charles A. Gersbach

**Affiliations:** 1 Department of Biomedical Engineering, Duke University, Durham, 27708 NC USA; 2 Center for Genomic and Computational Biology, Duke University, Durham, 27708 NC USA; 3 University Program in Genetics and Genomics, Duke University Medical Center, Durham, 27710 NC USA; 4 Division of Laboratory Animal Resources, Duke University School of Medicine, Durham, 27710 NC USA; 5 Department of Orthopaedic Surgery, Duke University Medical Center, Durham, 27710 NC USA

## Abstract

CRISPR-Cas9 transcriptional repressors have emerged as robust tools for disrupting gene regulation in vitro but have not yet been adapted for systemic delivery in adult animal models. Here we describe a *Staphylococcus aureus* Cas9-based repressor (dSaCas9^KRAB^) compatible with adeno-associated viral (AAV) delivery. To evaluate dSaCas9^KRAB^ efficacy for gene silencing in vivo, we silenced transcription of *Pcsk9*, a regulator of cholesterol levels, in the liver of adult mice. Systemic administration of a dual-vector AAV8 system expressing dSaCas9^KRAB^ and a *Pcsk9*-targeting guide RNA (gRNA) results in significant reductions of serum Pcsk9 and cholesterol levels. Despite a moderate host response to dSaCas9^KRAB^ expression, Pcsk9 repression is maintained for 24 weeks after a single treatment, demonstrating the potential for long-term gene silencing in post-mitotic tissues with dSaCas9^KRAB^. In vivo programmable gene silencing enables studies that link gene regulation to complex phenotypes and expands the CRISPR-Cas9 perturbation toolbox for basic research and gene therapy applications.

## Abstract (web summary)

Repression of gene transcription using CRISPR-Cas9 has been achieved in vitro but not for delivery into adult animal models. Here, the authors use AAV8 to deliver the transcriptional repressor dSaCas9^KRAB^ to the cholesterol regulator Pcsk9, and show repression up to 24 weeks and reduced cholesterol levels in mice.

## Introduction

Targeted gene regulation technologies enable interrogation of gene−phenotype relationships and strategies to guide cell function for applications such as genetic reprogramming and gene therapy^1,2^. The CRISPR-Cas9 platform has been widely applied for programmable gene regulation^3–10^. In this system, a deactivated, nuclease-null Cas9 (dCas9) acts as a DNA-binding domain that can be targeted by an engineered guide RNA (gRNA) molecule to bind any site in the genome containing a protospacer-adjacent motif (PAM)^6,11,12^. dCas9 can be genetically fused to various transcriptional or epigenetic modulators to modify gene expression in a customizable and site-specific manner^1,2^. dCas9 fusions to the Krüppel-associated box epigenetic repressor motif (dCas9^KRAB^) in particular have demonstrated widespread efficacy and a high degree of specificity for programmable gene silencing in cell culture models^4,5,13–15^.

Adapting targeted repressors for use in vivo would facilitate studies of gene regulation in complex organisms and the development of strategies to address aberrant gene regulation in disease. However, although various viral and non-viral methods have been explored for Cas9-mediated gene editing in animal models^16,17^, CRISPR/Cas9-based repressors have only been applied in mouse models by cell implantation after ex vivo modification^18^ or by direct injection of lentivirus into the brain, an invasive method resulting in localized gene delivery^19^. For gene regulation and therapeutic applications, adeno-associated viral (AAV) vectors are particularly advantageous as they have been extensively engineered to target a variety of tissue types and can be administered systemically^20,21^. Furthermore, AAVs also provide stable episomal gene expression with minimal integration, in contrast to lentiviral delivery, and are currently being tested clinically for a variety of gene therapy indications^20,21^. However, the AAV genome is limited to approximately 4.7 kilobases (kb) and the primary dCas9 variant used for gene regulation, *Streptococcus* *pyogenes* Cas9 (SpCas9), measures 4.2 kb. AAV-based delivery strategies with SpCas9 nuclease have demonstrated efficacy in vivo^22–24^, but RNA-guided gene regulators have an additional payload of the epigenetic effector module. Furthermore, surpassing AAV vector genome limits reduces viral titer and results in a heterogeneous viral pool containing truncated genomes^25,26^.

A smaller Cas9 nuclease derived from *Staphylococcus aureus* (SaCas9) has been used for genome editing in vivo in the liver^27^ and skeletal muscle^28,29^. The SaCas9 gene is 3.2 kb, and the corresponding PAM for SaCas9 is 5′-NNGRRT-3′, where N represents any nucleotide and R represents purine bases. We sought to adapt SaCas9 as a general tool for in vivo gene silencing that is compatible with delivery with AAV vectors. We generated a deactivated SaCas9-KRAB repressor (dSaCas9^KRAB^) and evaluated its efficacy as a transcriptional modulator in vitro and in vivo in wild-type adult mice. To demonstrate the potential of RNA-guided repressors to modulate clinically relevant targets, we used this approach to suppress transcription of the *Pcsk9* gene in adult wild-type mice. *Pcsk9* encodes an enzyme that regulates low-density lipoprotein (LDL) receptor degradation, and loss-of-function mutations in *Pcsk9* are associated with low serum cholesterol levels and reduced risk of cardiovascular disease with no recorded adverse side effects^30–32^. Strategies to inhibit expression of *Pcsk9* by antibodies, antisense oligonucleotides, and genome editing are currently being explored to lower harmful LDL cholesterol in the serum and reduce cardiovascular disease risk, with antibody-based therapies approved for treatment of familial hypercholesterolemia^27,33–37^. The mechanism and phenotypic effects of *Pcsk9* silencing are well understood, making this an advantageous model for testing the efficacy of in vivo silencing with CRISPR/Cas9 repressors.

To accommodate the limited packaging capacity of AAV, we designed a dual-vector system to deliver dSaCas9^KRAB^ and a single gRNA for targeted repression of an endogenous gene in vivo. We demonstrate transcriptional silencing of *Pcsk9* in the liver and reductions in secreted Pcsk9 and LDL cholesterol levels. Silencing effects are durable, as reductions in serum Pcsk9 are sustained long term after a single treatment with dSaCas9^KRAB^ and gRNA. These results establish the function of RNA-guided repressors in vivo, further expanding their utility as a technology to understand and modify gene regulation in development and disease.

## Results

### In vitro gene silencing with dSaCas9-based repressors

**Fig. 1:**
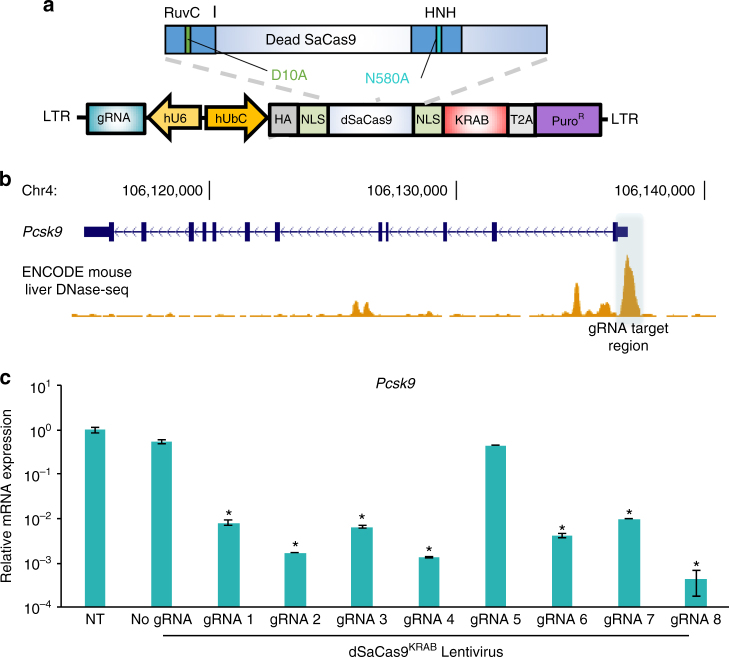
Targeted gene silencing of endogenous *Pcsk9* in vitro. **a** Deactivated *S. aureus* dCas9 was fused to a KRAB repressor motif and delivered by lentivirus for in vitro gRNA screening. The lentiviral vector also contained a puromycin resistance gene and a gRNA expression cassette. **b** A panel of eight *S. aureus* gRNAs were designed to target the accessible chromatin region of the mouse *Pcsk9* promoter region in AML12 cells, a mouse hepatocyte cell line with high expression of *Pcsk9*. An ENCODE wild-type mouse liver DNase I hypersensitivity-sequencing track is included to highlight the accessible chromatin region around the *Pcsk9* transcription start site^38^. **c** Single gRNAs were screened for silencing efficacy by qRT-PCR. (mean ± s.e.m., *n* = 2 biological replicates). *P* *<* 0.05 indicated by * compared with the non-transduced (NT) control (Student’s *t-*test)

While SaCas9 has been described as a nuclease for gene editing^27^, our goal in this study was to adapt SaCas9 for targeted gene silencing. We first sought to show that dSaCas9^KRAB^ could effectively silence genes in vitro. As a model, we harvested primary mouse fibroblasts from a mouse strain that constitutively expresses a luciferase reporter from a CAG promoter. We stably expressed dSaCas9^KRAB^ and gRNAs targeted to the CAG promoter by lentiviral transduction (Fig. 1a, Supplementary Fig 1a, Supplementary Table 1). Seven days after transduction, three of six promoter-targeting gRNAs significantly reduced luciferase expression compared to negative controls of untransduced cells and cells transduced with dSaCas9^KRAB^ but no gRNA (Supplementary Fig 1b, c).

For an endogenous gene target for in vivo studies, we selected *Pcsk9*, a regulator of LDL cholesterol levels targeted for repression in therapies for familial hypercholesterolemia. We screened several gRNAs for optimal repression of the mouse *Pcsk9* gene, our target for in vivo transcriptional repression (Supplementary Table 2). *Pcsk9* is highly expressed in the liver, and we designed gRNAs to target the DNase I hypersensitivity site surrounding the transcription start site in *Pcsk9* in adult mouse liver tissue (Fig. 1b)^38^. We tested these gRNAs in the AML12 mouse hepatocyte cell line. When delivered stably with dSaCas9^KRAB^ by lentiviral transduction, seven of eight gRNAs repressed *Pcsk9* transcript expression >90% by qRT-PCR, compared to non-treated controls and controls without a gRNA (Fig. 1c). These results demonstrate the effectiveness of RNA-guided SaCas9-based repressors for silencing target gene transcription in vitro.

### In vivo gene silencing in an adult wild-type mouse

**Fig. 2:**
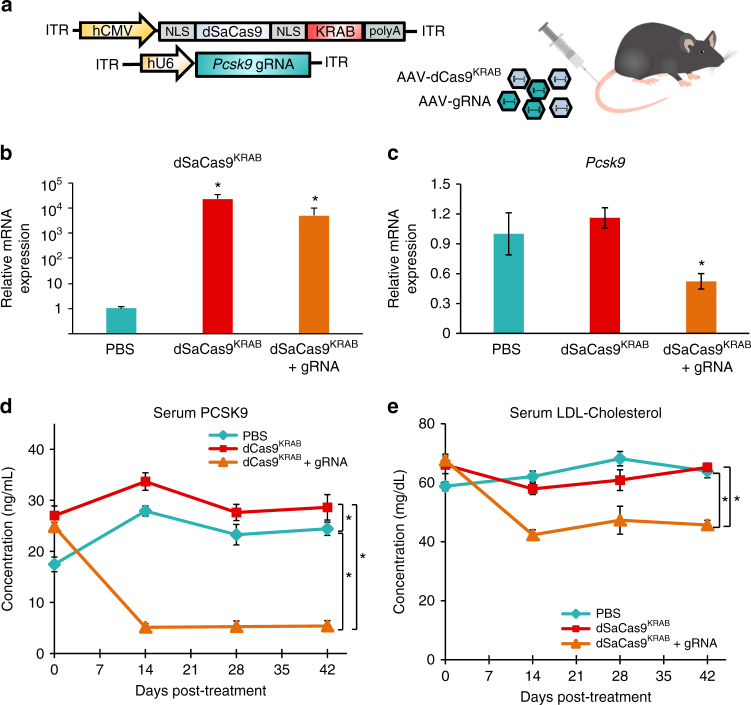
Targeted gene silencing in adult wild-type mice with *S. aureus* dCas9^KRAB^. **a** A dual AAV vector system was designed to deliver dSaCas9^KRAB^ and Pcsk9-targeting gRNA to adult-wild type mice via tail-vein injection. qRT-PCR for **b** dSaCas9^KRAB^ and **c** Pcsk9 expression was performed on mRNA from livers harvested from treated mice at 6 weeks post-injection (mean ± s.e.m, *n* = 4 mice). *P* *<* 0.05 is indicated by *, determined by Student’s *t-*test compared to controls. Serial serum collections were assayed for secreted **d** Pcsk9 protein levels and **e** low-density lipoprotein cholesterol (mean ± s.e.m., *n* = 4 mice, * indicates *P* < 0.05 by mixed design ANOVA with Tukey’s post-hoc analysis)

For targeted gene repression in vivo, we generated two AAV vectors, one encoding dSaCas9^KRAB^ (4.7 kb between inverted terminal repeats) and the other containing an expression cassette of the human U6 promoter driving a *Pcsk9*-targeting gRNA (4.2 kb between inverted terminal repeats including a stuffer sequence) (Fig. 2a). We selected *Pcsk9*-targeting gRNA 2 from our screen in AML12 cells for in vivo studies. We used two separate vectors to achieve high levels of dSaCas9^KRAB^ expression by including the full-length CMV promoter and the bGH-derived polyadenylation signal. Separating dSaCas9^KRAB^ and the gRNA on two vectors also maximized flexibility of experimental design and AAV production, including testing the effects of dSaCas9^KRAB^ and the gRNA independently.

We administered AAV to 6–8-week-old C57Bl/6 mice systemically by tail-vein injection using an AAV8 serotype to target hepatocytes in the liver that highly express *Pcsk9*. We tested two different doses of AAV expressing dSaCas9^KRAB^ and *Pcsk9*-targeting gRNA at 2×10^11^ and 1×10^12^ viral genomes per vector per mouse (vg/v/m). Age-matched controls received a PBS sham injection or dSaCas9^KRAB^ AAV injection without gRNA at 1×10^12^ vg/v/m. At 6 weeks post-treatment, we detected expression of dSaCas9^KRAB^ in the liver via qRT-PCR (Fig. 2b). Compared to PBS sham and dSaCas9^KRAB^-only controls, we observed significant transcriptional silencing of the *Pcsk9* gene in mouse livers treated with dSaCas9^KRAB^ and *Pcsk9*-targeting gRNA AAVs at a dose of 2×10^11^ vg/v/m (Fig. 2c). We collected serum from treated mice longitudinally to track Pcsk9 protein levels over time. Delivery of dSaCas9^KRAB^ and *Pcsk9*-targeting gRNA AAVs dramatically reduced Pcsk9 serum levels to 20% of levels in PBS-treated controls within 2 weeks of treatment (Fig. 2d). With the higher AAV dose of 1×10^12^ vg/v/m, Pcsk9 serum levels were reduced >90% over 4 weeks after treatment, but this magnitude of silencing was not sustained (Supplementary Fig. 2). In fact, at 6 weeks post-administration of the higher AAV dose, *Pcsk9* transcript levels were indistinguishable from non-treated controls and serum protein levels displayed an increasing trend relative to earlier time points. In contrast, *Pcsk9* silencing in mice receiving the lower dose of AAV was sustained throughout 6 weeks post-AAV delivery (Fig. 2d).

Concomitant with reduced Pcsk9 serum levels, we also observed significant reductions in serum levels of LDL and total cholesterol over 6 weeks post-treatment compared to PBS and dSaCas9^KRAB^ controls without the gRNA vector (Fig. 2e, Supplementary Fig. 3a). Reductions in LDL cholesterol serum levels were corroborated by western blot showing increases in LDL receptor expression in liver tissue expressing dSaCas9^KRAB^ and *Pcsk9* gRNA (Supplementary Fig. 3b). Together these results demonstrate that AAV delivery and CRISPR-mediated transcriptional silencing of *Pcsk9* in adult mice is sufficient to modulate downstream effects on cholesterol regulation.

### Host responses to CRISPR-based repression and AAV vectors

**Fig. 3:**
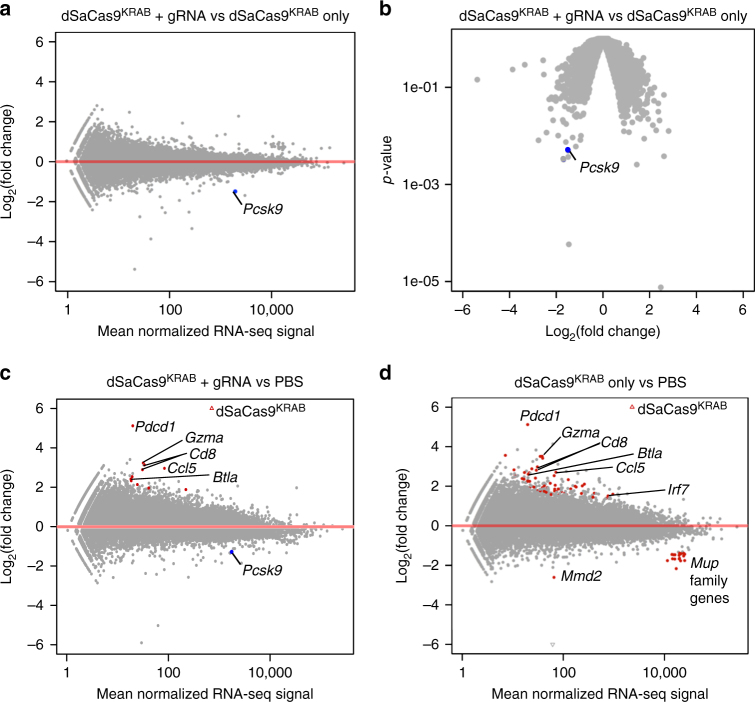
Genome-wide analysis of gene expression in dSaCas9^KRAB^-treated mice. **a**–**d** RNA-sequencing and differential expression analysis was performed comparing liver tissue from mice treated with **a**,** b** AAVs expressing dSaCas9^KRAB^ and *Pcsk9*-targeting gRNA vs. dSaCas9^KRAB^ alone, **c** AAVs expressing dSaCas9^KRAB^ with *Pcsk9*-targeting gRNA to PBS, or **d** AAV expressing dSaCas9^KRAB^ vs. PBS. Red data points indicate FDR < 0.05 by differential-expression analysis with a negative binomial generalized linear model and Wald test for significance^52^ (*n* = 4, mice 6–8 weeks old at time of treatment). The data point representing the *Pcsk9* transcript is highlighted in blue (FDR > 0.05). Extended gene lists found in Supplementary Data 1, 4, 5

We next investigated the genome-wide effects of CRISPR-mediated gene silencing on transcriptional regulation. We performed RNA-sequencing on mouse liver tissue at 6 weeks post-treatment. When comparing treatment with dSaCas9^KRAB^ and *Pcsk9* gRNA to dSaCas9^KRAB^ without the gRNA vector, no gene expression changes achieved genome-wide significance (defined by a false discovery rate, FDR < 0.05) (Fig. 3a, b). However, when ranking genes by raw *P-*value, *Pcsk9* repression was the seventh most significant transcriptional change genome-wide (Fig. 3a, b, Supplementary Data 1).

Previous studies performed with *S. pyogenes* dCas9^KRAB^ have demonstrated that CRISPR-guided dCas9^KRAB^ binding and gene regulation can be highly specific for the target gene locus in cell culture systems^15^. To explore potential off-target gene regulation effects with RNA-guided dSaCas9^KRAB^ silencing in vivo, we identified the top 100 differentially regulated genes by *p-*value when comparing liver tissue from mice treated with dSaCas9^KRAB^ and *Pcsk9*-targeting gRNA compared to dSaCas9^KRAB^ alone (Supplementary Data 1). We also computationally predicted 159 potential off-target binding sites for the *Pcsk9*-targeting gRNA in the mouse genome^39^, selected based on the presence of a 6 bp PAM-proximal seed sequence and fewer than ten total mismatches to the cognate target sequence^40^ (Supplementary Data 2). Of the top 100 differentially expressed genes identified, only one gene, *Sox1ot*, contained a predicted off-target binding site for the *Pcsk9*-targeting gRNA. However, *Sox1ot* expression was upregulated 1.9-fold in dSaCas9^KRAB^ and gRNA-treated mice, suggesting this gene regulation effect is not a direct result of dSaCas9^KRAB^ transcriptional repression. To explore the possibility of distal effects of dSaCas9^KRAB^ binding, we matched each of the top 100 differentially expressed genes to the closest computationally predicted off-target site of the *Pcsk9*-targeting gRNA (Supplementary Data 3). With the exceptions of *Pcsk9* and *Sox1ot*, none of the top 100 genes contained a predicted off-target site within 100 kilobases of the gene body. These results suggest that the top gene regulation changes observed in this study are not a function of off-target gRNA-mediated binding of dSaCas9^KRAB^ and may instead reflect downstream responses to AAV transduction and transgene expression or *Pcsk9* silencing and cholesterol reductions in a complex, heterogeneous tissue.

We also compared gene expression by RNA-sequencing in liver tissue treated with dSaCas9^KRAB^ and *Pcsk9* gRNA to PBS-treated controls. Here, we observed significant enrichment of nine genes (FDR < 0.05), including a subset specific to immune cells (*Btla*, *Cd8*, *Ccl5*, *Gzma*, *Irf7*, and *Pdcd1*), suggesting immune cell infiltration (Fig. 3c, Supplementary Data 4). Similar immune gene enrichment has been observed in recent studies with SpCas9-based activators delivered to skeletal muscle^41^. Delivery of dSaCas9^KRAB^ AAV alone without gRNA AAV was also sufficient to cause immune gene enrichment when compared to PBS controls (Fig. 3d, Supplementary Data 5).

To understand the contributions the dSaCas9^KRAB^ protein and *Pcsk9*-targeted gRNA in generating a host response, we compared mice treated with dSaCas9^KRAB^ AAV only, gRNA AAV only, and an equal mixture of two AAVs expressing dSaCas9^KRAB^ and gRNA at 4×10^11^ vg/v/m. Treatment with dSaCas9^KRAB^ and gRNA resulted in reduced Pcsk9 and LDL cholesterol serum in mice compared to controls within 2 weeks post-treatment (Supplementary Fig. 4). Immune cell-specific gene enrichment occurred primarily in response to dSaCas9^KRAB^ expression, indicating that this effect is independent of gRNA expression or *Pcsk9* repression (Supplementary Fig. 5, Supplementary Data 6−9). Widespread expression of genes related to immune response was accompanied by attenuation in Pcsk9 silencing at the transcriptional level (Supplementary Fig. 5b, Supplementary Data 6−9) and at the protein level in serum over 6 weeks post-treatment (Supplementary Fig. 4). These results highlight the importance of tuning AAV doses for dSaCas9^KRAB^ and gRNA delivery in order to maximize silencing effect and mitigate immune response.

**Fig. 4:**
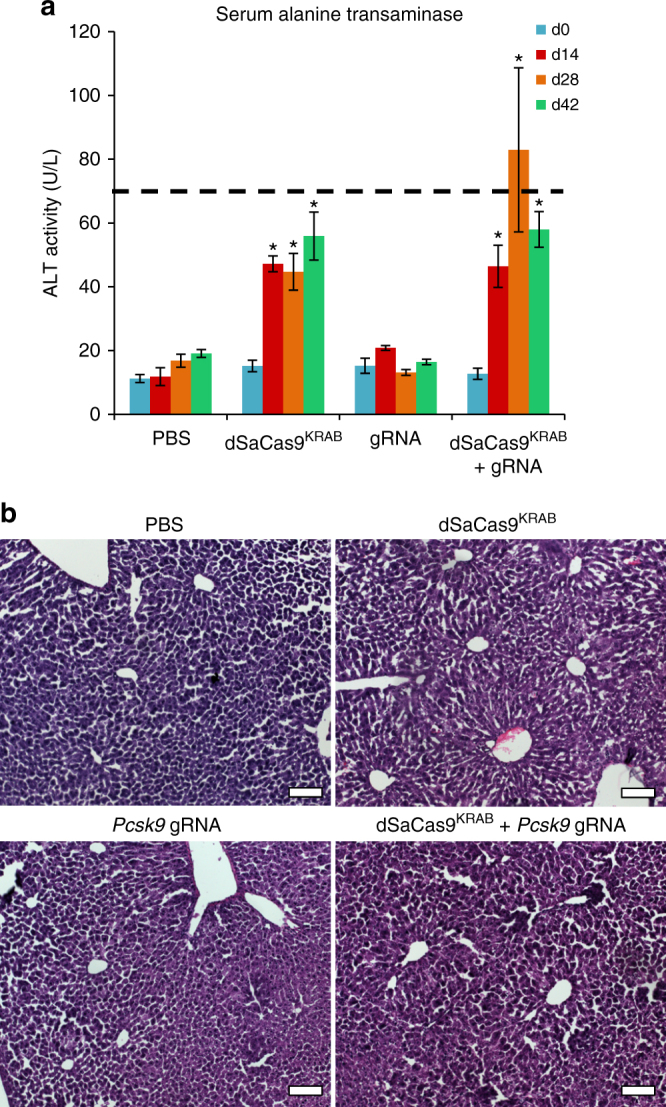
Transient effects of AAV-mediated dSaCas9^KRAB^ gene silencing on host liver. **a** Secreted alanine transaminase as a measure of liver toxicity was assayed in the serum of treated mice over 6 weeks post-treatment (mean ± s.e.m., *n* = 4 mice at 6–8 weeks old when treated). *P* < 0.05 is indicated by * and calculated by mixed design ANOVA with Tukey’s post-hoc analysis compared to day 0 controls. Dotted line marks the upper limit of physiological levels of ALT in adult C57Bl/6 mice. **b** H&E staining was performed on sections from livers of mice harvested 6 weeks post-treatment (representative images shown, scale bars = 100 μm)

To further investigate consequences of AAV delivery of dSaCas9^KRAB^ and the *Pcsk9*-targeted gRNA, we measured secretion of alanine transaminases (ALT). Elevated ALT secretion is a marker of liver toxicity and increases in ALT serum levels have been observed with AAV-mediated transgene expression targeted to the liver^42^. In this study, we detected increased ALT serum levels at 4 weeks after AAV administration, with up to a sixfold increase in mice treated with AAV-dSaCas9^KRAB^ compared to either PBS injection or the gRNA AAV alone (Fig. 4a). Despite these increases, ALT levels were within physiological ranges for male C57Bl/6 mice for all conditions at 6 weeks post-treatment. Furthermore, we observed similar normal tissue morphology across livers isolated from PBS-treated controls, gRNA-only controls, and dSaCas9^KRAB^-treated mice at 6 weeks after treatment (Fig. 4b). Together, these studies show that the host response, indicated by enrichment in immune cell gene expression and concurrent increase in ALT levels, are primarily a result of dSaCas9^KRAB^ expression and not simply the AAV capsid proteins.

### Long-term efficacy of in vivo transcriptional repression

**Fig. 5:**
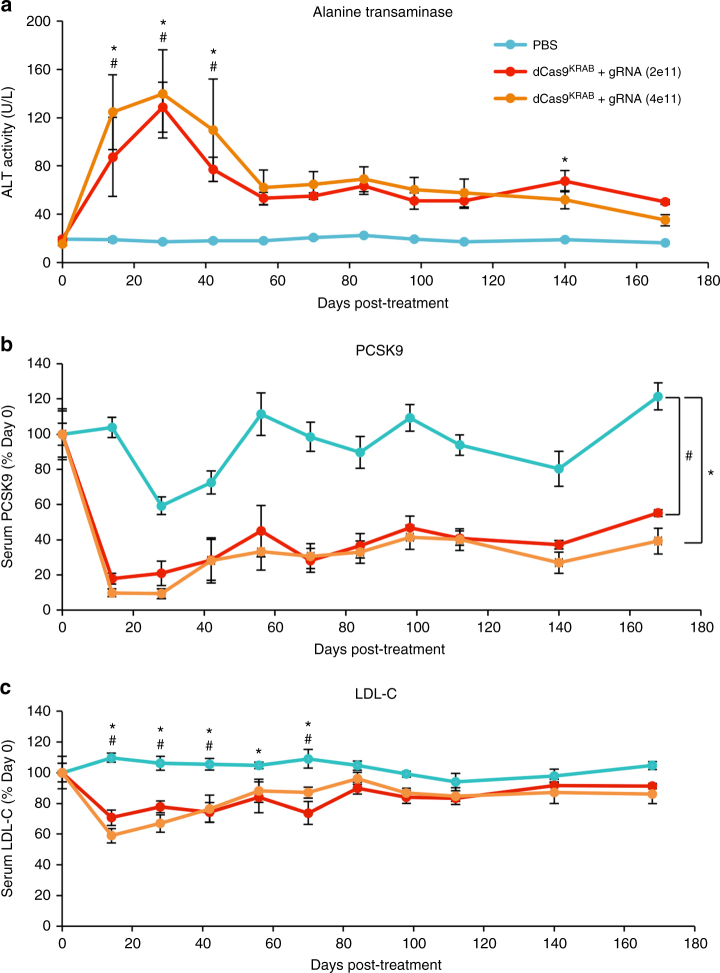
Long-term efficacy of targeted transcriptional silencing with dSaCas9^KRAB^. In mice treated with AAVs expressing dSaCas9^KRAB^ and *Pcsk9*-targeting gRNA at 2×10^11^ and 4×10^11^ viral genomes/vector/mouse, serum was collected over 168 days post-treatment and analyzed for **a** alanine transanimase secretion, **b** Pcsk9 protein concentration, and **c** low-density lipoprotein-cholesterol concentration (mean ± s.e.m., *n* = 4 mice at 6–8 weeks old when treated). * and # mark *P* < 0.05 in 2e11 and 4e11 doses respectively, calculated by mixed design ANOVA with Tukey’s post-hoc analysis compared to PBS controls

We tracked the long-term efficacy of dSaCas9^KRAB^-based silencing in vivo to further assess its usefulness as a research or gene therapy tool. We treated mice with two doses of AAVs expressing dSaCas9^KRAB^ and *Pcsk9*-targeted gRNA (2×10^11^ and 4×10^11^ vg/v/m) and collected serum over 24 weeks post-treatment. We measured ALT serum levels as an indication of how well this treatment was tolerated over time. The acute elevation of ALT levels we observed in our shorter-term studies dissipated past 6 weeks post-treatment and stabilized to levels within physiological ranges for the duration of the study, similar to what has been observed in clinical studies^43^ (Fig. 5a). This suggests that despite an initial toxicity response to AAV dSaCas9^KRAB^ expression, long-term liver function is not acutely compromised.

Notably, we observed durable dSaCas9^KRAB^-mediated silencing of Pcsk9 expression, with both doses maintaining significantly reduced serum Pcsk9 levels up to 24 weeks post-treatment compared to PBS-treated controls (Fig. 5b). Similar to ALT levels, we observed the most acute effects early after AAV injection followed by an attenuation and then stabilization of repression. In mice treated with 4×10^11^ vg/v/m of dSaCas9^KRAB^ and *Pcsk9* gRNA AAVs, we measured up to 90% repression of Pcsk9 levels until 4 weeks after treatment, after which Pcsk9 was repressed to 27–41% of day 0 levels for the duration of the study. Reduction in LDL-cholesterol levels was sustained through 10 weeks post-treatment, suggesting potential compensatory effects for cholesterol regulation in response to *Pcsk9* repression at later timepoints (Fig. 5c). Overall, these results demonstrate that RNA-guided dSaCas9^KRAB^ repressors are capable of durable, long-term target gene silencing in vivo.

## Discussion

The robustness and specificity of CRISPR-Cas9 gene regulation has shown great promise for applications in gene therapy and studying gene function^1^. Here we present a strategy to translate RNA-guided gene repression to a wild-type mouse model via AAV delivery and demonstrate that dSaCas9^KRAB^ functions effectively in vivo. AAV8 delivery of dSaCas9^KRAB^ and gRNA to the liver of adult wild-type mice resulted in potent and durable silencing of Pcsk9 expression and associated reductions in serum cholesterol.

Gene regulation with CRISPR-Cas9 is typically achieved with stable expression of the gRNA and dCas9 fusion protein^1^, in contrast to transient expression strategies often employed with nuclease-based genome editing^17^. AAV gene delivery provides long-term episomal expression with minimal genomic integration and is well-suited for gene regulation applications targeting post-mitotic tissues with low cellular turnover, including liver, heart, skeletal muscle, the central nervous system, and ocular tissues.

For in vivo gene repression with dCas9^KRAB^, we used SaCas9 to account for the size constraint of AAV packaging. However, two recent studies have also reported in vivo transcriptional modulation with AAV and SpCas9-based activators^41,44^. In one approach, intein peptides were incorporated to dimerize split modules of a SpCas9-activator independently expressed from two AAVs, leading to modest short-term activation of target gene expression upon delivery to neonatal mice^41^. The other study employed dual AAVs expressing a full-length SpCas9 and a gRNA modified to recruit the transactivation complex using an MS2−aptamer interaction^44^. Intramuscular delivery to neonatal mice resulted in phenotypic effects concurrent with transcriptional activation. We have built upon this work by demonstrating delivery of CRISPR-Cas9 components and transcriptional modulation long term in adult mice. One potential benefit of our dSaCas9^KRAB^ system is that we only need to deliver two components, minimizing the additional recruitment steps that could reduce efficiency or foreign peptides that may contribute to an immune response.

Our findings highlight the importance of assessing and mitigating host immune responses for CRISPR-Cas9 gene regulation. Similar to studies using SaCas9 nuclease and SpCas9-based activators in vivo^27,28,41^, we observed some host responses to AAV-mediated expression of the foreign SaCas9 protein but the effects on liver function were moderate in nature and did not compromise gene expression or tissue morphology. However, the loss of silencing we observed within 6 weeks of AAV administration may be due in part to increased cellular turnover in the liver due to tissue damage associated with host response, as suggested by elevated ALT secretion levels and enrichment in immune gene expression in treated liver tissue. When we directly compared a 2×10^11^ vs. 1×10^12^ vg/v/m dose, the lower dose treatment sustained greater efficacy over 6 weeks, and in long-term studies, we achieved sustained Pcsk9 repression over 24 weeks with our lowest dose tested, 2×10^11^ vg/v/m. These results support that tuning the expression of CRISPR-Cas9 components to achieve a desired magnitude of gene expression and phenotypic response may be critical to the longevity and tolerability of targeted gene regulation in vivo. Mitotically stable, ‘hit-and-run’ epigenome editing approaches^45^ or combined treatment with immunomodulatory drugs could also lead to further improvements for applications that require long-term gene regulation.

This platform can be customized to target a tissue of interest by selecting particular AAV serotypes or tissue-restricted promoters for dSaCas9^KRAB^. Any gene or regulatory element can be targeted for regulation by switching the gRNA protospacer sequence. Finally, the genomic target can be modified in a variety of ways by selecting from several available epigenome editing effectors^1^. These studies establish the potential of the CRISPR-Cas9 system for dissecting gene regulation mechanisms, understanding disease, and modulating gene expression for therapeutic applications in complex organisms.

## Methods

### Plasmid constructs and AAV design

An inactive version of SaCas9 (dSaCas9) was created by introducing D10A and N580A mutations^27^. We used a *S. aureus* Cas9 AAV expression plasmid (Addgene #61592)^46^ and replaced the nuclease-active SaCas9 with dSaCas9^KRAB^. We also removed the C-terminal 3x HA epitope tag and incorporated a single N-terminal HA tag for tracking protein expression. The AAV and lentivirus plasmids expressing dSaCas9^KRAB^ are available on Addgene (Addgene #106249 and #106219). To screen gRNAs, we cloned an expression cassette for the SaCas9 gRNA^27^ expressed from the human U6 promoter upstream of the human UbC promoter in the lentiviral vector via *Pac*I sites. To create the AAV-gRNA plasmid, a Pcsk9 *S. aureus* gRNA cassette expressed from a human U6 promoter was cloned into an AAV stuffer plasmid (Addgene #106248) at the KpnI and XbaI sites, replacing a CMV promoter.

### Cell culture

AML12 cells and HEK293T cells were obtained from the American Tissue Collection Center (ATCC) through the Duke University Cancer Center Facilities. Cell lines were verified via morphological inspection and were not tested for mycoplasma. AML12 cells were maintained in DMEM/F12 supplemented with 10% FBS and 1% penicillin-streptomycin. HEK293T cells were cultured in DMEM supplemented with 10% FBS and 1% penicillin-streptomycin. Primary fibroblasts were obtained from 4–6-week-old male FVB-Tg(CAG-luc,-GFP)L2G85Chco/J (Jackson Labs) mice and cultured on collagen-coated plates in DMEM supplemented with 10% FBS and 1% penicillin-streptomycin. All cells were cultured at 37 °C with 5% CO_2_.

### Lentiviral transduction

AML12 cells and primary fibroblasts were transduced with lentivirus to stably express dSaCas9^KRAB^ and *Pcsk9-*targeting gRNA. To produce VSV-G pseudotyped lentivirus, HEK293T cells were plated at a density of 4e6 cells in 10-cm dishes. Cells were co-transfected by calcium phosphate precipitation with dSaCas9^KRAB^ and gRNA lentiviral expression plasmids (20 μg), packaging plasmid psPAX2 (Addgene #12260, 15 μg), and envelope plasmid pMD2.G (Addgene #12259, 6 μg)^47^. A media change was performed the next morning, and medium containing lentivirus was collected 24 and 48 h later. Lentiviral supernatant was cleared of remnant cells by filtration through 0.45 μm cellulose acetate filters and concentrated 20-fold into PBS using Lenti-X Concentrator solution (Clontech). Concentrated viral supernatant was snap-frozen and stored at −80 °C for future use. For transduction, concentrated viral supernatant was diluted 1 to 20 in the culture medium of the cells being transduced, with addition of the cationic polymer polybrene at 4 μg/mL to facilitate transduction. Non-transduced (NT) cells did not receive virus but were treated with polybrene as a control. The day after transduction, the medium was exchanged. A puromycin dose of 1 μg/mL puromycin was used for selection approximately 96 h after transduction.

### AAV production

AAV vectors were tested for potential recombination and integrity of the inverted terminal repeat by SmaI digest of the transfer plasmid before production. AAV-dSaCas9^KRAB^ and AAV-U6 Pcsk9 gRNA were used to generate AAV8 in two separate batches by the Gene Transfer Vector Core at Schepens Eye Research Institute, Massachusetts Eye and Ear.

### Animal studies

Animal studies were conducted with adherence to the guidelines for the care and use of laboratory animals of the National Institutes of Health (NIH). All the experiments with animals were approved by the Institutional Animal Care and Use Committee (IACUC) at Duke University. Each study was performed with *n* = 4 male *C57Bl/6* mice (Jackson Labs) at 6–8 weeks of age per group, determined from pilot studies as a minimum number to account for technical and biological variability in the experiment. Mice were anesthetized and maintained at 37 °C. Within an experiment, animals aged matched by date of birth were assigned randomly to a treatment group and injections were blinded to treatment group. The tail vein was prepared and injected with 200 µL of AAV solution (2×10^11^−2×10^12^ viral genomes/total dose) or sterile PBS using a 31 G needle. Mice were injected with a saline control, AAV-dSaCas9^KRAB^ alone, AAV-U6 Pcsk9 gRNA alone, or a 1:1 dose mixture of AAV-dSaCas9^KRAB^ and AAV-U6 Pcsk9 gRNA. Mice were fasted for 12–14 h and submandibular vein blood collections were performed every 2 weeks, starting on day 0 four to six hours prior to tail vein injection. Mice were euthanized by CO_2_ inhalation, perfused with PBS, and tissue was collected into RNALater^®^ (Life Technologies) for DNA and RNA, snap-frozen for protein analysis, or fixed in 4% PFA and embedded in OCT for histology.

### qRT-PCR

Tissue samples were stored in RNALater (Ambion) and total RNA was isolated using the RNA Universal Plus Kit (Qiagen). cDNA synthesis was performed using the SuperScript VILO cDNA Synthesis Kit (Invitrogen). Quantitative real-time PCR (qRT-PCR) using QuantIT Perfecta Supermix was performed with the CFX96 Real-Time PCR Detection System (Bio-Rad) with the oligonucleotide primers and reaction conditions optimized for 90–110% amplification efficiency (Supplementary Table 3). The results are expressed as fold-increase mRNA expression of the gene of interest normalized to *Gapdh* expression by the ΔΔ*C*_*t*_ method.

### RNA-sequencing

mRNA was purified from total RNA using oligo(dT) Dynabeads (Invitrogen). SuperScript VILO cDNA Synthesis Kit (Invitrogen) was used for first-strand cDNA synthesis, followed by the second-strand cDNA reaction using DNA polymerase I (New England Biolabs). DNA purifications were performed using Agencourt AMPure XP beads (Beckman Coulter). cDNA was fragmented and ligated with sequencing primers by treatment with Nextera transposase (Illumina) for 5 min at 55 °C. Transposase activity was quenched by QG buffer (Qiagen), followed immediately by bead purification of tagmented DNA. Indexed sequencing libraries were PCR-amplified and sequenced for 50-bp paired-end reads on an Illumina HiSeq 2000 instrument at the Duke Genome Sequencing Shared Resource. Reads that aligned to the delivered AAV vector were removed from analysis from treated samples. Reads were aligned to the mouse RefSeq transcriptome using Bowtie2^48^. Statistical analysis, including multiple hypothesis testing, on four independent biological replicates was performed using a negative binomial distribution model in DESeq^49^. We identified genes linked to immune response using Gene Set Enrichment Analysis software^50^, supplemented by manual inspection. Off-target site prediction for the *Pcsk9*-targeting gRNA was conducted using Cas-OFFinder^39^ and matched to potential downstream genes through the Bedtools suite “closest” function^51^.

### Western blot

Minced tissue was lysed in RIPA buffer (Sigma), and total protein quantified via the BCA assay (Pierce). Lysates were mixed with LDS sample buffer (Invitrogen) and boiled for 5 min; equal amounts of total protein were run in NuPAGE Novex 4–12% Bis-Tris polyacrylamide gels (Life Technologies) and transferred to nitrocellulose membranes. Non-specific antibody binding was blocked with 5% nonfat milk in TBS-T (50 mM Tris, 150 mM NaCl and 0.1% Tween-20) for 30 min. The membranes were then incubated with primary antibody in 5% milk in TBS-T: rabbit anti-LDLR (Abcam Clone EP1553Y) diluted 1:1000 overnight at 4 °C or rabbit anti-GAPDH (Cell Signaling Clone 14C10) diluted 1:5000 for 60 min at room temperature. Membranes labeled with primary antibodies were incubated with anti-rabbit HRP-conjugated antibody (Sigma-Aldrich, A6154) diluted 1:5000 for 60 min and washed with TBS-T for 60 min. Membranes were visualized using the Immun-Star WesternC Chemiluminescence Kit (Bio-Rad) and images were captured using a ChemiDoc XRS+ system and processed using ImageLab software (Bio-Rad). Uncropped western images are shown in Supplementary Fig. 6.

### Histology

A cross section of the median liver lobe was fixed overnight in 4% PFA and embedded in OCT using liquid nitrogen-cooled isopentane. 10 µm sections were cut onto pre-treated histological slides. Hematoxylin and eosin was used to reveal general liver histopathology.

### Serum analysis

After harvest, serum was stored in one-time use aliquots at −80 °C. Total cholesterol and LDL cholesterol levels were measured from serum via a colorimetric assay according to the manufacturer’s instructions (ThermoScientific Total Cholesterol Reagents #TR13421 and WakoChemical LDL Cholesterol #993–00404). ALT secretion was measured from serum via a colorimetric assay according to the manufacturer’s instructions (Sigma-Aldrich #MAK052). Pcsk9 serum protein levels were quantified by ELISA with a standard curve according to the manufacturer’s instructions (R&D Systems #MPC900).

### Data availability

Raw RNA-seq data are available on Gene Expression Omnibus (GSE109608).

## Electronic supplementary material

Supplementary Information

Description of Additional Supplementary Files

Supplementary Data 1–9
