# Supplementary material for: RNA-guided transcriptional silencing in vivo with S. aureus CRISPR-Cas9 repressors
Source: Nat Commun. 2018 Apr 26;9:1674. doi: 10.1038/s41467-018-04048-4 (PMC5920046; doi:10.1038/s41467-018-04048-4)
Supplement: Supplementary file 1 — Supplementary Information [file 41467_2018_4048_MOESM1_ESM.pdf]

RNA-Guided Transcriptional Silencing *In Vivo* with *S. aureus* CRISPR-Cas9 Repressors

Thakore et al.

Supplementary Information

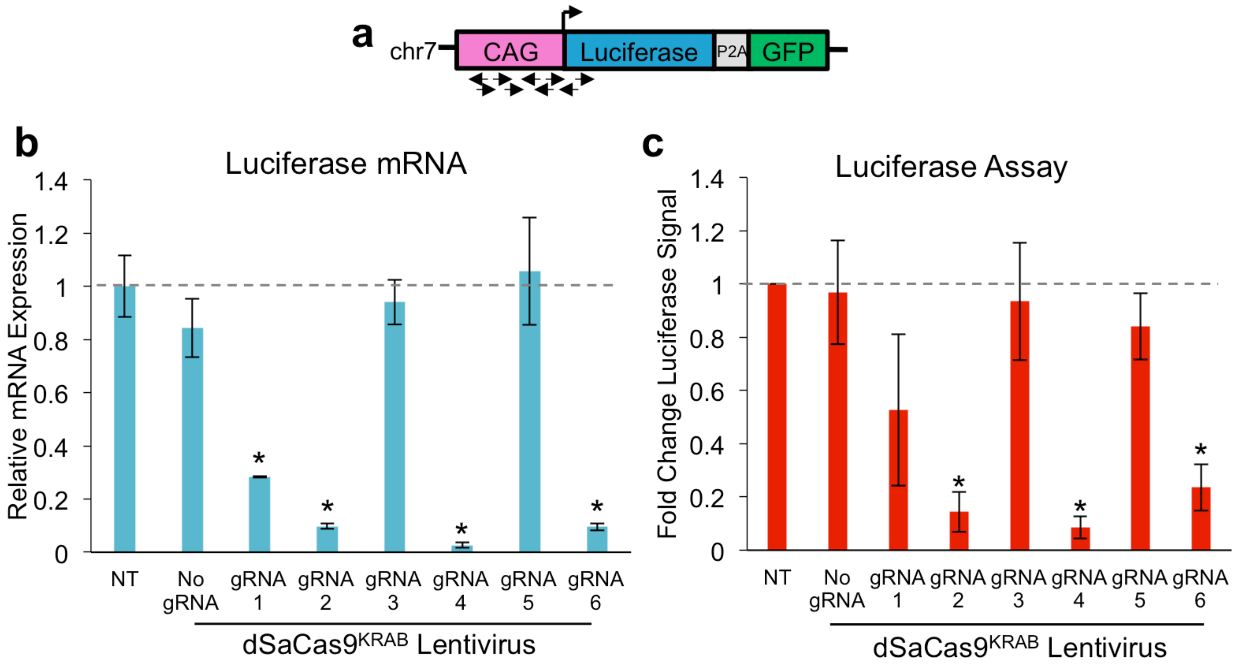

Supplementary Figure 1. Targeted transcriptional modulation *in vitro* with dSaCas9^{KRAB}. (a) Deactivated *S. aureus* Cas9 was fused to a KRAB repressor motif (dSaCas9^{KRAB}) and delivered by lentivirus for *in vitro* gRNA screening. The lentiviral vector also contained a puromycin resistance gene and a gRNA expression cassette. (b) Single gRNAs were designed to target the CAG promoter driving luciferase expression in mouse primary fibroblasts. Silencing efficacy for dSaCas9^{KRAB} and gRNAs was evaluated by (c) qRT-PCR for the luciferase transcript and (d) luciferase assay (mean ± s.e.m., $n = 2$ biological replicates). *$P < 0.05$ compared with the non-transduced (NT) control (Student's *t*-test).

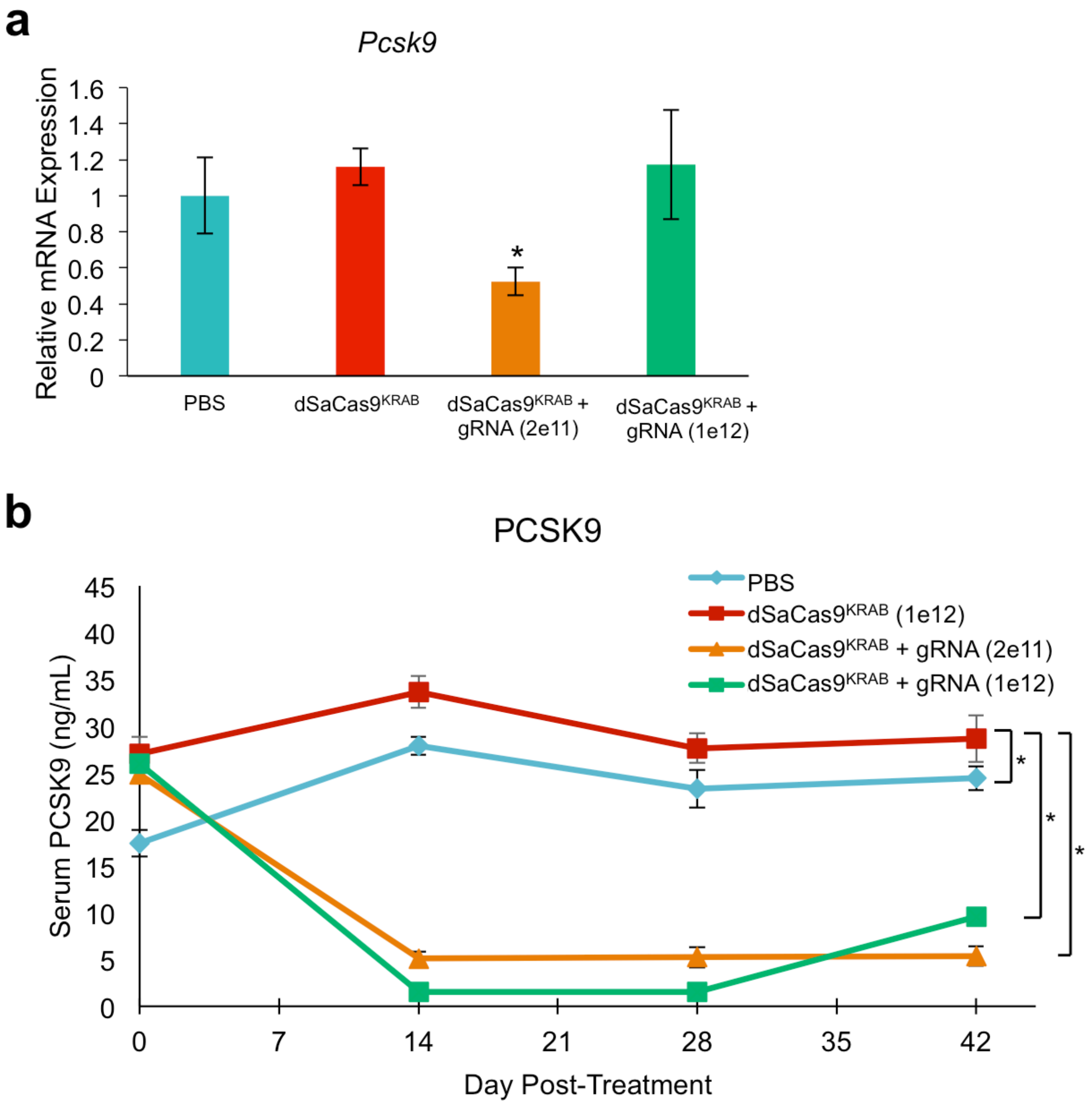

Supplementary Figure 2. Evaluation of high and low dose AAV for targeted gene silencing in adult wild-type mice with dSaCas9^{KRAB}. (a) qRT-PCR for *Pcsk9* expression was performed on livers harvested from 6-8 week old mice treated with AAV at the indicated doses (viral genomes/vector/mouse) at 6 weeks post-injection (mean ± s.e.m, n = 4 mice). * indicates $p < 0.05$ by Student's t-test compared to controls). **(b)** Serial serum collections were assayed for secreted PCSK9 protein (mean ± s.e.m , n = 4 mice). * indicates $P < 0.05$ by mixed design ANOVA with Tukey's post-hoc analysis.

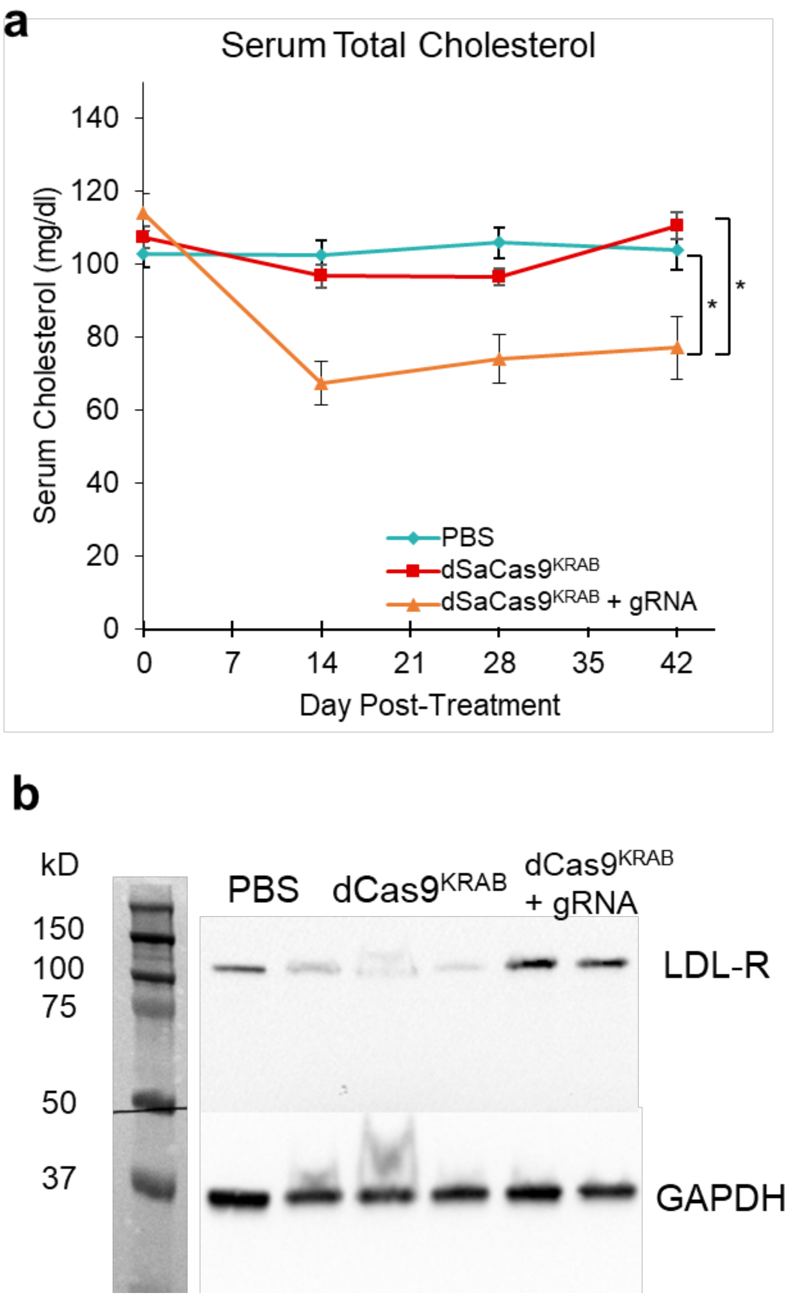

Supplementary Figure 3. Effects of targeted transcriptional silencing of *Pcsk9* on cholesterol regulation. (a) Total cholesterol was measured from serum collected every two weeks from treated mice (mean ± s.e.m, n = 4 mice, * indicates $P < 0.05$ by mixed design ANOVA with Tukey's post-hoc analysis compared to PBS and dCas9-KRAB only controls). **(b)** Livers were assayed for LDL-receptor expression by Western blot 6 weeks post-treatment. Raw, uncropped membrane images shown in **Supplementary Fig 6**.

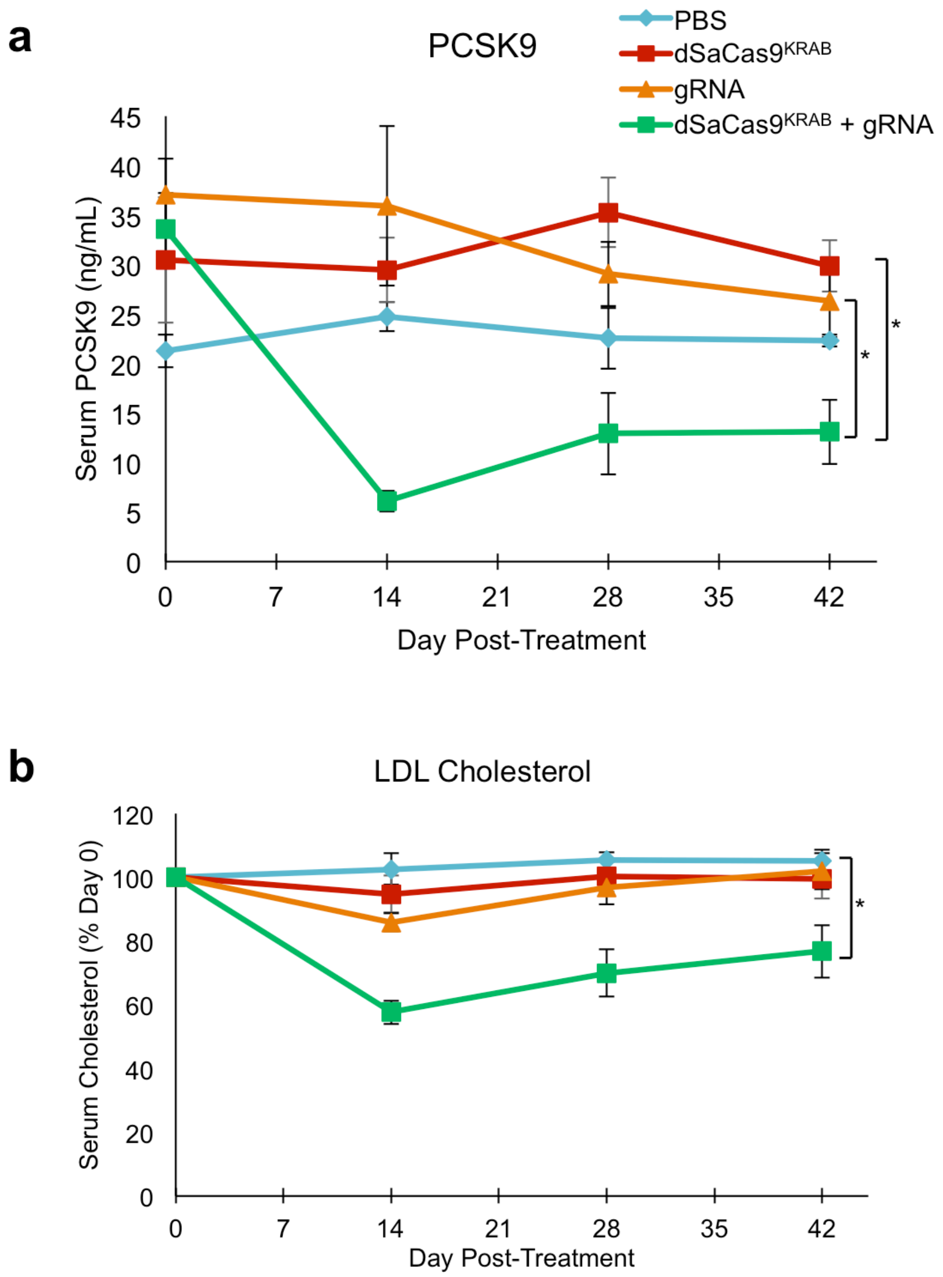

Supplementary Figure 4. Targeted *Pcsk9* repression in adult wild-type mice treated with AAVs expressing dSaCas9^{KRAB} and gRNA at 4e11 viral genomes/vector/mouse. (a) PCSK9 concentration and **(b)** LDL cholesterol were measured from serum collected every two weeks from treated mice (mean ± s.e.m, n = 4 mice). * indicates $P < 0.05$ by mixed design ANOVA with Tukey's post-hoc analysis compared to indicated control.

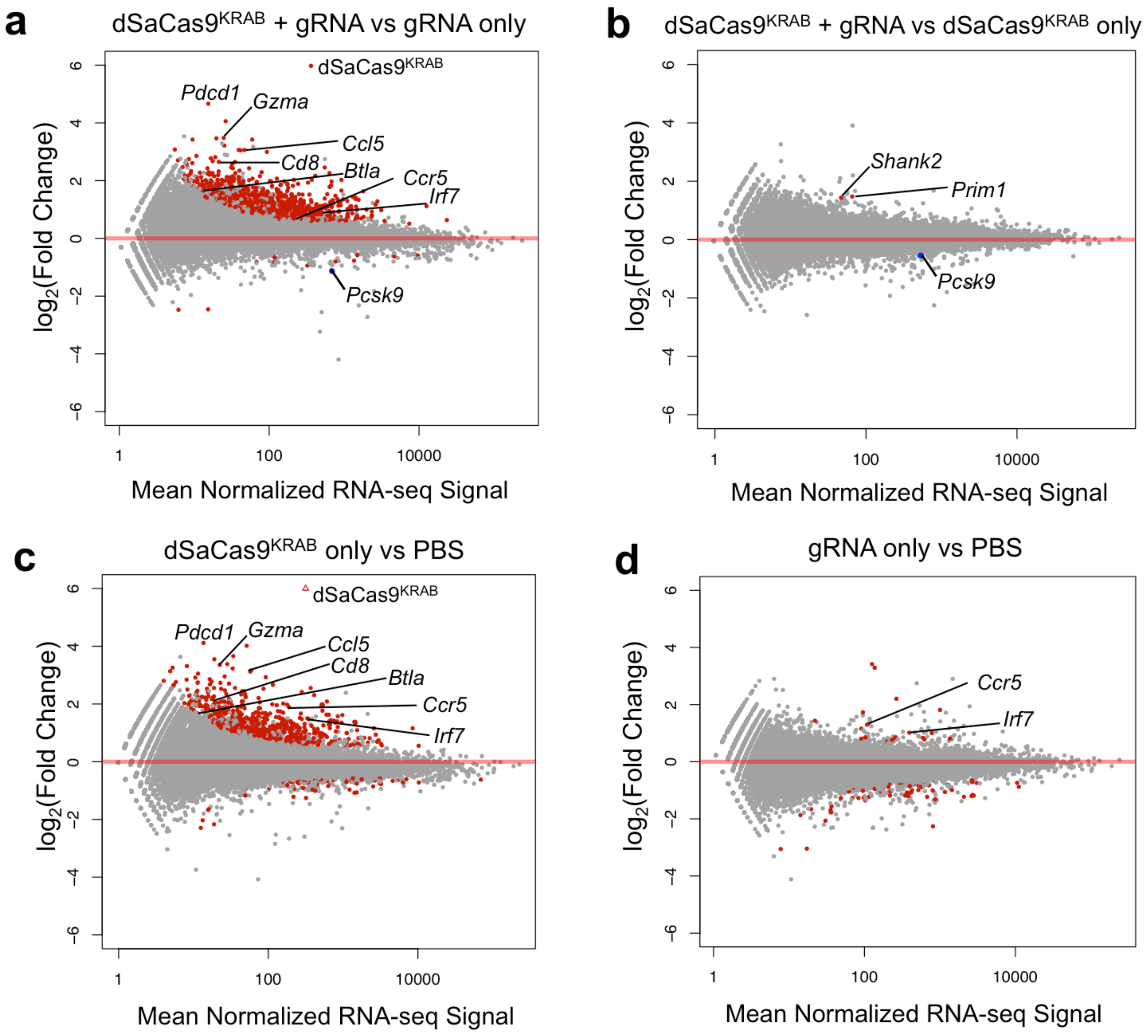

Supplementary Figure 5. Genome-wide analysis of gene expression in liver tissue from mice treated with AAVs expressing dSaCas9^{KRAB} and *Pcsk9*-targeting gRNA show enrichment of immune cell genes in the presences of dSaCas9^{KRAB}. (a,b) Differential expression analysis comparing liver tissue from mice treated with **(a)** AAVs expressing dSaCas9^{KRAB} and *Pcsk9*-targeting gRNA versus gRNA alone, **(b)** AAVs expressing dSaCas9^{KRAB} and *Pcsk9*-targeting gRNA versus dSaCas9^{KRAB} alone, **(c)** AAVs expressing dSaCas9^{KRAB} versus PBS, and **(d)** AAVs expressing *Pcsk9*-targeting gRNA to PBS. Red data points indicate false discovery rate, FDR < 0.05 by differential expression analysis using a negative binomial model with the Wald test for significance (n = 4 mice). The data point presenting the Pcsk9 transcript is highlighted in blue (FDR > 0.05). Extended gene lists provided in **Supplementary Data 6-9**.

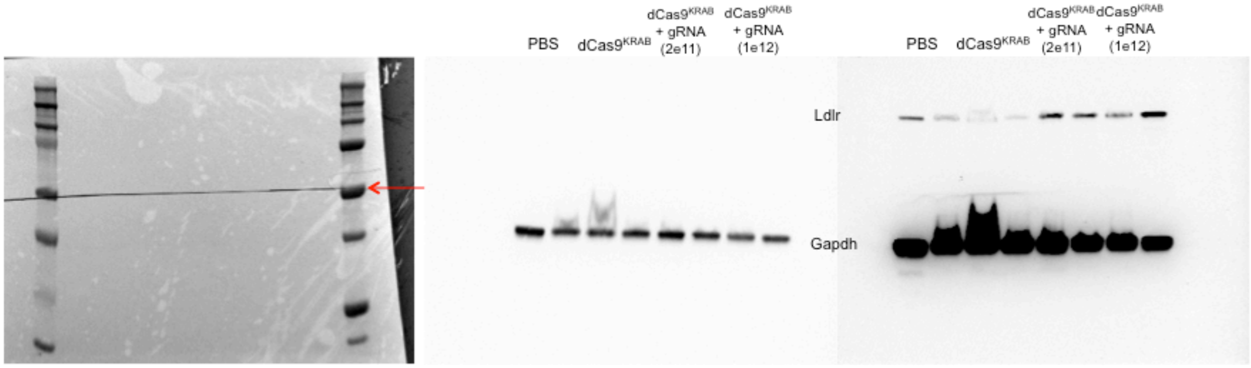

Supplementary Figure 6. Ldlr protein expression with *Pcsk9* silencing. Uncropped images of Ldlr and Gapdh western blots in livers from mice treated with dSaCas9^{KRAB} with Pcsk9 gRNA at two doses (viral genomes/vectors/mouse) show an increase in Ldlr expression compared to dSaCas9^{KRAB} only and PBS controls. Before primary antibody incubation, the membrane was cut (red arrow) and the top half was treated with anti-LDLR antibody, while the bottom half was treated with anti-GAPDH antibody. The two images show increasing exposure time to capture Gapdh (left) and Ldlr (right) expression.

Supplementary Table 1. Panel of CAG promoter-targeting gRNA protospacer target sequences.

gRNA #	Protospacer	PAM	Strand
1	gtcattattgacgtcaatgggc	GGGGGT	-
2	gtgctcagcaactcggggag	GGGGGT	-
3	ctcggggagggggggtgcagg	GGGGGT	-
4	actttccattgacgtcaatggg	TGGACT	+
5	cttcggggggggacggggcaggg	CGGGGT	+
6	cttcgccccgcgcccgctaga	GGGGGT	-

Supplementary Table 2. Panel of *Pcsk9*-targeting gRNA protospacer target sequences.

gRNA #	Protospacer	PAM	Strand
1	ggcatctttgaagatttaaatg	TGGAGT	+
2	gagggaagggatacaggctgga	TGGAGT	+
3	gaagtcggcactccacgcagtg	AAGAGT	+
4	gcactccacgcagtgaagagtc	ATGGGT	+
5	gccccacctctttagcatttc	CGGGGT	-
6	ggcaccccgcctgaggaggat	TGGAGT	+
7	gtttgcagcccaattaggatt	TGGGGT	+
8	gagaggatcttccgatggggct	CAGGGT	+

Supplementary Table 3. Primer sequences used for qPCR

Target	Primer
qPCR *Gapdh* Fwd	CCTCGTCCCGTAGACAAAATG
qPCR *Gapdh* Rev	TGAAGGGGTCGTTGATGGC
qPCR *Pcsk9* Fwd	GCTTCTGCTCCAGAGGTCAT
qPCR *Pcsk9* Rev	CTCCGATGATGTCCTTCCCG
qPCR *dSaCas9* Fwd	CGAGCGGATCGAGGAAATCA
qPCR *dSaCas8* Rev	GCTTCACGAGCACCTTGTTG
