# Supplementary material for: RNA-guided transcriptional silencing in vivo with S. aureus CRISPR-Cas9 repressors
Source: Nat Commun. 2018 Apr 26;9:1674. doi: 10.1038/s41467-018-04048-4 (PMC5920046; doi:10.1038/s41467-018-04048-4)
Supplement: Supplementary file 2 — Description of Additional Supplementary Files [file 41467_2018_4048_MOESM2_ESM.docx]

**Description of Additional Supplementary Files**

File Name: Supplementary Data 1

Description:

Top 100 genes with significantly altered gene expression by p-value for RNA-sequencing of liver tissue from mice treated with AAV vectors expressing dSaCas9KRAB and Pcsk9-targeting gRNA compared to AAV expressing dSaCas9KRAB alone by DESeq.

File Name: Supplementary Data 2

Description:

Computationally predicted off-target sites for Pcsk9-targeting gRNA. We used the Cas-OFFinder tool to computationally predict potential off-target binding sites of Pcsk9-targeting gRNA in the mouse genome. We selected off-target sites based on the following criteria: fewer than 10 total mismatches to the target sequence and containing an exact match to a 6 base-pair “seed” sequence adjacent to an SaCas9-compatible 5`-NNGRRT-3` PAM. Lowercase lettering in the sequence column indicates a mismatch to the Pcsk9 target gRNA sequence.

File Name: Supplementary Data 3

Description:

Closest predicted Pcsk9-gRNA off-target binding site to top 100 differentially regulated genes from livers treated with AAVs expressing dSaCas9KRAB and Pcsk9-targeting gRNA compared to dSaCas9KRAB-only controls.

File Name: Supplementary Data 4

Description:

Genes with significantly altered gene expression by RNA-sequencing of liver tissue from mice treated with AAV vectors expressing dSaCas9KRAB and Pcsk9-targeting gRNA compared to PBS-treated controls. Genes are ranked by false discovery rate, FDR < 0.05. Gene associated with immune response are bolded.

File Name: Supplementary Data 5

Description:

Genes with significantly altered gene expression by RNA-sequencing of liver tissue from mice treated with AAV expressing dSaCas9KRAB compared to PBS-treated controls. Genes are ranked by false discovery rate, FDR < 0.05. Gene associated with immune response are bolded.

File Name: Supplementary Data 6

Description:

Top 100 genes with significantly altered gene expression by RNA-sequencing of liver tissue from mice treated with AAV vectors expressing dSaCas9KRAB and Pcsk9-targeting gRNA compared to an AAV-Pcsk9 gRNA control at 4e11 viral genomes/vector/mouse. Genes are ranked by false discovery rate, FDR < 0.05. Gene associated with immune response are bolded.

File Name: Supplementary Data 7

Description:

Top 100 genes with significantly altered gene expression by RNA-sequencing of liver tissue from mice treated with AAV vectors expressing dSaCas9KRAB and Pcsk9-targeting gRNA compared to an AAV-dSaCas9KRAB control at 4e11 viral genomes/vector/mouse. Genes are ranked by false discovery rate, FDR < 0.05. Gene associated with immune response are bolded.

File Name: Supplementary Data 8

Description:

Genes with significantly altered gene expression by RNA-sequencing of liver tissue from mice treated with AAV expressing Pcsk9-targeting gRNA at 4e11 viral genomes/vector/mouse compared to a PBS control. Genes are ranked by false discovery rate, FDR < 0.05. Genes associated with immune response are bolded.

File Name: Supplementary Data 9

Description:

Top 100 genes with significantly altered gene expression by RNA-sequencing of liver tissue from mice treated with AAV expressing dSaCas9KRAB at 4e11 viral genomes/vectors/mouse compared to a PBS control. Genes are ranked by false discovery rate, FDR < 0.05. Genes associated with immune response are bolded.
